# Supplementary material for: Identifying profiles of stressors and stress management strategies in Filipinos: A secondary analysis
Source: Glob Ment Health (Camb). 2026 Apr 6;13:e77. doi: 10.1017/gmh.2026.10192 (PMC13125274; doi:10.1017/gmh.2026.10192)
Supplement: Fudolig et al. supplementary material [file S2054425126101927sup001.docx]

**Supplementary Table S1: Latent Class Analysis Fit Indices for 2-6 classes.**

| **#Classes** | **BIC** | **MLE Exist?** | **AIC** | **Rel. Entropy** | **LMR (p-value)** |
| --- | --- | --- | --- | --- | --- |
| 2 | 11674 | Yes | 11587 | 0.544 | 199 (<0.001) |
| 3 | 11662 | Yes | 11523 | 0.507 | 78.5 (<0.001) |
| 4 | 11630 | Yes | 11452 | 0.615 | 84.9 (<0.001) |
| 5 | 11664 | Yes | 11438 | 0.636 | 30.8 (<0.001) |
| 6 | 11691 | No | 11433 | 0.696 | 22.0 (0.009) |
